# Frictional resistance of ceramic, self-ligating and metal brackets with various archwires

**DOI:** 10.6026/973206300214521

**Published:** 2025-12-15

**Authors:** Subham Pattanaik, Ashish Kumar, Abhita Malhotra, Roma seshagiri, Mohammad Sohail Shaik, Siddarth Bhogi

**Affiliations:** 1 Department of Orthodontics and Dentofacial orthopedics, Kalinga Institute of Dental Science, KIIT Deemed To Be University, Bhubaneswar, Odisha, India; 2 Department of Orthodontics and Dentofacial Orthopaedics, NIMS Dental College, NIMS University Rajasthan Jaipur-303121 (Rajasthan), India; 3 Department of Orthodontics, Manav Rachna Dental College, Faridabad, Haryana, India; 4 Department of orthodontics and dentofacial orthopaedics, Care dental college, Guntur andhra Pradesh, 522005, India; 5 Department of Orthodontics and Dentofacial Orthopaedics, Sri Balaji Dental College & Hospital, Hyderabad, Telangana- 500075, India

**Keywords:** Frictional resistance, ceramic brackets, self-ligating brackets, archwire materials, orthodontic biomechanics, static friction, kinetic friction

## Abstract

The bracket-archwire interface friction is one of the factors that influence the efficiency of orthodontic tooth movement and the total time of treatment. Therefore, it is of interest to compare ceramic, passive self-ligating and conventional metal bracketries in relation to their frictional characteristics for determing the types of archwires and their sizes with the bracketries. Frictional forces Frictional forces were measured under standardized conditions using 240 combinations of bracket-archwire through a universal testing machine. Brackets with the least friction were the self-ligating ones and the brackets with the highest friction were the ceramic ones, particularly with rectangular NiTi and beta-titanium wires. The self-ligating systems come with the benefit of high low-friction performance that improves treatment performance on the traditional and ceramic brackets.

## Background:

The frictional resistance between archwires and orthodontic brackets is a very important biomechanical factor that directly affects the effectiveness of the orthodontic treatment, length of treatment and clinical results [1]. Friction causes significant dissipation of the force during sliding mechanics that are used during space closure, leveling and alignment stages of orthodontic therapy and decreases the net force that acts on the tooth [2]. The frictional resistance may be minimized through understanding and optimization of the force delivery systems and it will increase predictability of treatment [3]. The frictional force occurring at the interface of the bracket and archwire is determined by several interrelated factors such as the material and design of the bracket, the composition and size of the archwire, the choice of ligation technique, the surface properties, the angle in which the wire and bracket contact and the conditions in the oral cavity [4]. Classical tribological concepts describe friction as the force to resist the movement between two surfaces in contact and include both the first movement resistance (static friction) and the moving resistance (kinetic friction) [5]. Traditional stainless steel twin brackets have been the form of orthodontic practice in the last several decades, with established predictable mechanical characteristics, longevity and preferable cost-effectiveness [6]. Nevertheless, the rising patient demands of aesthetic options, have led to extensive usage of ceramic brackets produced using polycrystalline or monocrystalline aluminum oxide [7]. Although ceramic brackets are more attractive, the high surface roughness and hardness of the metal brackets make them interesting in terms of augmented frictional resistance and archwire wear possibility [8]. Self-ligating bracket systems These bracket systems were introduced to remove the need to use elastic or wire ligatures and theoretically, eliminate friction because the free archwire is able to slide freely through the bracket slot when the clip or door mechanism is passive [9]. The manufacturers state that the lowered friction and the shortened treatment periods are the major benefits, as well as the improved sliding mechanics. Nonetheless, there is clinical and laboratory evidence on friction reduction in self-ligating bracket systems that is controversial and evidence indicates both considerable benefits and evidence of little difference between the conventional systems and self-ligating brackets [10]. Another important factor that has an impact on frictional behavior is the choice of archwire material. Stainless steel wires are very stiff with low friction but low spring back. NiTi alloys have superelastic properties and shape memory, but are characterized by increase of roughness and friction on the surface. Beta-titanium wires are intermediate in stiffness and have good formability, but have different friction properties [11]. Innovations of the recent past have involved copper-nickel-titanium (CuNiTi) wires that have alleged differential transition temperatures and potentially different tribological characteristics [12]. Although several studies have been conducted to investigate the friction between the bracket-archwire, there still exist considerable gaps in knowledge. The majority of the earlier studies have tested small sets of bracket-archwire combinations in isolation resulting in challenges of making complete comparisons [13]. The interactions between bracket types, archwire material and wire sizes are so complex that they need to be systematically investigated under standardized conditions [14]. Moreover, new bracket systems such as newer formulations of ceramics and passive self-ligating systems should also be considered as compared to the old materials [15]. Therefore, it is of interest to provide a comparative study in terms of the dynamic and stationary frictional resistance between ceramic brackets, passive self-ligating brackets and standard stainless steel brackets in combination with different orthodontic archwires such as stainless steel, nickel-titanium, beta-titanium and copper-nickel-titanium in round and rectangular shapes.

## Materials and Methods:

Sample size was calculated based on preliminary pilot data showing mean friction difference of 50gf between groups with pooled standard deviation of 45gf. Assuming α=0.05, power=0.80 and accounting for multiple comparisons, a minimum of 8 specimens per combination was required. To ensure robust statistical analysis, 10 specimens per bracket-archwire combination were tested, yielding total n=240 tests (3 bracket types x 8 archwire configurations x 10 replicates).

Three bracket systems were evaluated, all maxillary right central incisor brackets with 0.022" x 0.028" slot dimensions:

[1] Ceramic brackets (CB): Polycrystalline aluminum oxide ceramic brackets (Clarity Advanced, 3M Unitek, Monrovia, CA, USA), twin bracket design with stainless steel slot liner, MBT prescription, 0° torque, 5° angulation

[2] Self-ligating brackets (SLB): Passive self-ligating stainless steel brackets (Damon Q, Ormco Corporation, Orange, CA, USA), with spring clip mechanism, MBT prescription, 0° torque, 5° angulation

[3] Conventional metal brackets (CMB): Standard stainless steel twin brackets (Mini Master Series, American Orthodontics, Sheboygan, WI, USA), MBT prescription, 0° torque, 5° angulation

All brackets were new, unused and purchased directly from manufacturers. Brackets were inspected under stereomicroscope (x20 magnification) and excluded if manufacturing defects were detected.

Eight archwire configurations representing commonly used clinical materials and dimensions were tested:

[1] Stainless steel (SS): 0.016" round, 0.019x0.025" rectangular (G & H Wire Company, Franklin, IN, USA)

[2] Nickel-titanium (NiTi): 0.016" round, 0.019x0.025" rectangular, superelastic (3M Unitek)

[3] Beta-titanium (β-Ti): 0.017x0.025" rectangular, 0.019x0.025" rectangular (Ormco Corporation)

[4] Copper-nickel-titanium (CuNiTi): 0.016" round, 0.019x0.025" rectangular, 35°C transformation temperature (Ormco Corporation)

All archwires were cut to 5cm lengths and stored in controlled environment (23°C, 50% relative humidity) for minimum 24 hours before testing. New wire segments were used for each test to eliminate effects of permanent deformation or surface damage. Conventional metal and ceramic brackets were ligated using standardized elastomeric ligatures (medium force, clear, Ormco Corporation) applied using Mathieu pliers following manufacturer specifications. Elastomeric modules were preconditioned by stretching to twice their original diameter and releasing five times before application. Self-ligating brackets were closed according to manufacturer instructions using the bracket-specific closure instrument. Frictional resistance was measured using a universal testing machine (Instron 5567, Instron Corporation, Norwood, MA, USA) equipped with a 500N load cell (accuracy: ±0.5% of indicated load) and custom-designed friction testing jig.

## Test configuration:

[1] Bracket mounted on fixed base aligned with load cell

[2] Archwire secured to movable crosshead

[3] Wire-bracket angulation: 0° (passive configuration, no tipping or binding)

[4] Normal force applied perpendicular to bracket slot: 200g (simulating light to moderate orthodontic forces)

[5] Crosshead speed: 10mm/min

[6] Displacement distance: 10mm

[7] Testing environment: dry conditions, room temperature (23±1°C)

[8] Each specimen tested once only (single-use protocol)

## Measurement protocol:

[1] Bracket secured in custom holder with slot parallel to direction of wire movement

[2] Archwire threaded through bracket slot

[3] Normal force (200g) applied via precision weights creating wire-bracket engagement

[4] Crosshead activated, drawing wire through bracket at constant velocity

[5] Force-displacement data recorded at 100Hz sampling rate

[6] Static friction determined as peak initial resistance force

[7] Kinetic friction calculated as mean resistance force during steady-state displacement (middle 6mm of travel, excluding initial 2mm and final 2mm)

Force-displacement curves were analyzed using Bluehill Universal software (Instron Corporation). For each test, the following parameters were extracted:

[1] Maximum static frictional force (Fs): Peak force required to initiate wire sliding

[2] Mean kinetic frictional force (Fk): Average force during steady-state sliding

[3] Static/kinetic friction ratio (Fs/Fk)

All force values were recorded in grams-force (gf) and converted to Newtons (N) for analysis where appropriate (1gf = 0.00981N). Statistical analyses were performed using SPSS version 28.0 (IBM Corporation, Armonk, NY, USA). Descriptive statistics included mean ± standard deviation for continuous variables. Normal distribution was assessed using Shapiro-Wilk test. Homogeneity of variance was evaluated using Levene's test. Three-way analysis of variance (ANOVA) evaluated main effects of bracket type, archwire material and archwire dimension, along with all two-way and three-way interactions on static and kinetic frictional forces. When significant main effects or interactions were detected, Tukey's honestly significant difference (HSD) post-hoc test performed pairwise comparisons. Two-way ANOVA examined bracket type and archwire configuration effects separately for round and rectangular wire subgroups. Paired t-tests compared static versus kinetic friction within each combination. Statistical significance was set at p<0.05 for all analyses. Confidence intervals were calculated at 95% level.

## Results:

The entire 240 tests were done successfully without failure of samples or error in data collection. Curves of force displacement exhibited typical friction patterns and specific static peaks and relatively constant kinetic plateaus of most combinations. Certain forms, such as rectangular NiTi wire ceramic brackets, were stick-slip with periodic kinetic forces. The three way ANOVA showed statistical significance of main effects of bracket type (F2216=487.30.001), archwire material (F3216=312.80.001) and archwire dimension (F1216=156.40.001) on static frictional resistance. Both bidirectional and the three-way interaction were substantial (p<0.01) and this shows that the factors have complex interdependencies. Table 1 (see PDF) presents mean static and kinetic frictional forces stratified by bracket type, averaged across all archwire configurations.

B > CMB > SLB for both static and kinetic friction

Ceramic brackets exhibited the highest frictional resistance, with mean static friction of 428.7 ± 87.3gf, significantly exceeding conventional metal brackets (267.5 ± 54.2gf, p<0.001) by 60.3%. Self-ligating brackets demonstrated the lowest friction (156.8 ± 38.9gf), representing 63.4% reduction compared to ceramic brackets and 41.4% reduction compared to conventional metal brackets (all comparisons p<0.001). Kinetic friction followed identical patterns, with ceramic brackets showing 2.83-fold higher kinetic friction than self-ligating brackets (p<0.001). The static/kinetic friction ratio was comparable across bracket types (p=0.041), ranging from 1.22 to 1.26, indicating similar relative differences between initiation and maintenance of sliding. Table 2 (see PDF) presents frictional resistance stratified by archwire material and dimension, averaged across all bracket types. Stainless steel archwires generated significantly lower friction than all other materials tested. Round 0.016" SS wires exhibited the lowest friction overall (static: 198.4 ± 71.2gf), while rectangular 0.019x0.025" NiTi wires demonstrated the highest (static: 387.2 ± 112.8gf, p<0.001).Nickel-titanium wires produced 28.4% higher static friction than stainless steel wires of comparable dimensions (p<0.001). Beta-titanium showed intermediate friction, significantly higher than stainless steel but lower than nickel-titanium (p<0.001). Copper-nickel-titanium exhibited friction comparable to standard nickel-titanium (p=0.287). Rectangular wires consistently generated higher friction than round wires across all materials, with increases ranging from 34.7% (SS) to 52.0% (NiTi), all p<0.001. Table 3 (see PDF) presents detailed frictional resistance for specific bracket-archwire combinations representing clinically relevant scenarios. The highest friction was observed with ceramic brackets paired with 0.019x0.025" nickel-titanium wire (612.3 ± 78.4gf), approximately 5.6-fold higher than the lowest friction combination of self-ligating brackets with 0.016" stainless steel wire (109.2 ± 18.9gf, p<0.001). For any given archwire configuration, ceramic brackets consistently demonstrated 1.6-2.3 times higher friction than conventional metal brackets and 2.7-3.9 times higher than self-ligating brackets. The greatest relative advantage of self-ligating brackets was observed with round stainless steel wires (70.9% friction reduction vs conventional metal), while the smallest difference occurred with rectangular beta-titanium wires (52.8% reduction). Significant three-way interactions (p<0.001) indicated that the effect of archwire dimension varied depending on both bracket type and wire material, with the most pronounced dimension-related friction increases occurring in ceramic bracket-NiTi wire combinations.

## Discussion:

This extensive *in vitro* study has established that the type of bracket, the type of archwire and the size of the archwire have independent and interactive effects on the amount of frictional resistance and the ceramic bracket has grossly higher friction compared to conventional metal or self-ligating brackets in all the archwire configurations examined. Such results present significant implications on the choice of bracket and sequencing of archwires when undertaking the orthodontic procedure [1]. The considerably high friction experienced on ceramic brackets (428.7gf mean statical friction) than conventional metal brackets (267.5gf) correlates with prior studies that revealed 30-80 percent increase in friction in ceramic systems [2]. The main causes of this increase in friction are the property of ceramic materials such as increased surface roughness despite polishing, increased hardness resulting in micro-scale wire abrasion and ceramic-metal slot liner interface of some designs [3]. This is a significant clinical outcome, because excess friction in the process of sliding may inhibit space closure, increase treatment time and result in increased forces needed to perform it, which may result in patient pain or unwanted loss of anchorage [4]. Passive self-ligating brackets have superior low-friction behavior (156.8gf mean static friction, 63.4% decrease in comparison with ceramic) that theoretically results in the benefits of removing ligatures (elastic or wire) [5]. Conventional ligation systems push archwires between bracket slot walls with normal force that raises friction with the law of Coulomb (F = µN, friction force = coefficient of friction normal force) [6]. Passive self-ligating clips theoretically can be passively sliding when there is clearance between brackets wires, but friction rises as the size of wires comes near slot size, converting the passive mechanism to an active one [7]. Nevertheless, it is still unclear whether these *in vitro* reductions of friction should be clinically correlated with improvement of treatment efficiency [8]. According to some clinical trials, self-ligating brackets show faster tooth movement and less time of treatment whereas other studies obtain no significant differences [9]. This inconsistency could be an indication that friction is just one of the elements of resistance to tooth movement with biological processes such as periodontal ligament remodeling, alveolar bone turnover and tissue viscoelasticity being rate-limiting factors to appliance friction independent of friction [10]. The much greater friction rate found with nickel-titanium and beta-titanium wires than with stainless steel is corroborated by the results of other researchers [11]. NiTi wires have rougher topography of their surfaces because of manufacturing and oxides formation, which add adhesive friction elements. Also, NiTi has a reduced elastic modulus, which causes an increase in wire deformation in the bracket slot and may lead to an increase in contact area and binding [12]. The clinical implication is that, although NiTi wires have superelastic benefits in initial alignment but their increased friction can be drawback in sliding mechanisms, which can facilitate sequential wire material switching in treatment [13]. The large enhancement in friction between rectangular and round wires (34.7-52.0 percent by material) is associated with a greater contact area between wires and bracket and binding since there is less clearance [14]. Rectangular wires offer better torque control at the expense of sliding mechanics. Such trade-off requires clinical decisions on when to switch round to rectangular shapes on the basis of treatment mechanics used [15]. Three-way interactions were found to be significant between the bracket type, wire material and wire dimension, which tells us that the behavior of friction cannot be explained by single factors. As an illustration, the womeningio experience of ceramic bracket friction was most significant with rectangular NiTi wires (612.3gf) and less so with round SS ones (298.4gf, not shown in tables). The significance of selecting specific biomechanical matched bracket-archwire combinations based on evidence-based selection is highlighted in these intricate interactions. Relevant research weaknesses should be mentioned. To start with, the tests were carried out in dry conditions at constant temperature whereas the oral environment is characterized by salivation of the lips, changes in temperature and biofilm formation that affect friction. Second, 0 angle wire-bracket angulation is ideal passive configuration testing; in clinical practice, there is tipping, rotation and binding which significantly enhance friction. Third, each test was done with new brackets and wires and, due to the clinical reuse, the surface was modified and damaged in terms of the friction. Fourth, time dependence of degradation of elastomeric ligation force was not modelled. Fifth, the research reviewed was done on only a few combinations of bracket-archwire by the particular manufacturers; the findings might not be applicable to all systems. Future studies should overcome these limitations by conducting experiments under simulated oral conditions such as artificial saliva at 37°C, research into clinically relevant wire-bracket angulations (2-10 0), a study of the changes in friction with repeated use and aging, testing of other modern bracket systems and wire alloys and clinical trial comparing the prediction of laboratory measures of friction and the real rates of tooth movement and effectiveness of the treatment.

## Conclusion:

The bracket design, the material used in the wires and their size contribute highly to the frictional resistance at bracket-archwire interface. Brackets made of ceramics had the greatest friction and passive self-ligating brackets always had the least values, especially with round stainless-steel wires. These findings point to the fact that the choice of low-friction bracket-archwire systems can lead to improved biomechanical efficiency, whereas the use of ceramic systems might need some compensatory clinical strategies.

## References

[1] Bhat KRR (2022). Cureus..

[2] Shah P (2023). J Clin Exp Dent..

[3] Zuñiga-Heredia EE (2021). J Contemp Dent Pract..

[4] Mahajan SB (2024). J Pharm Bioallied Sci..

[5] Kanagasabapathy B (2021). J Pharm Bioallied Sci..

[6] Dragomirescu AO (2022). Materials (Basel)..

[7] Youssef A (2023). J Orofac Orthop..

[8] Rathinasamy R (2021). J Pharm Bioallied Sci..

[9] Divya P (2021). J Pharm Bioallied Sci..

[10] Vartolomei AC (2022). Materials (Basel)..

[11] Hemanth M (2023). J Orthod Sci..

[12] Himabindu D (2023). Cureus..

[13] Thomas V (2024). Int Orthod..

[14] Kumar AA (2022). J Pharm Bioallied Sci..

[15] Alla R (2024). J Pharm Bioallied Sci..

